# Evaluation of antibody-based single cell type imaging techniques coupled to multiplexed imaging of N-glycans and collagen peptides by matrix-assisted laser desorption/ionization mass spectrometry imaging

**DOI:** 10.1007/s00216-023-04983-2

**Published:** 2023-10-16

**Authors:** Jaclyn Dunne, Jake Griner, Martin Romeo, Jade Macdonald, Carsten Krieg, Mark Lim, Gargey Yagnik, Kenneth J. Rothschild, Richard R. Drake, Anand S. Mehta, Peggi M. Angel

**Affiliations:** 1 Department of Cell and Molecular Pharmacology & Experimental Therapeutics, Medical University of South Carolina, 173 Ashley Avenue BSB 358, Charleston, SC 29425 USA; 2 Translational Science Laboratory, Hollings Cancer Center, Charleston, SC 29425 USA; 3 Department of Pathology and Laboratory Medicine, Medical University of South Carolina, Charleston, SC 29425 USA; 4 AmberGen, Inc, 44 Manning Road, Billerica, MA 01821 USA; 5 Department of Physics and Photonics Center, Boston University, Boston, MA 02215 USA

**Keywords:** Multimodal imaging, Mass spectrometry imaging, Extracellular matrix, Single cell spatial analysis, Collagen peptides, N-Glycans

## Abstract

The integration of matrix-assisted laser desorption/ionization mass spectrometry imaging (MALDI-MSI) with single cell spatial omics methods allows for a comprehensive investigation of single cell spatial information and matrisomal N-glycan and extracellular matrix protein imaging. Here, the performance of the antibody-directed single cell workflows coupled with MALDI-MSI are evaluated. Miralys™ photocleavable mass-tagged antibody probes (MALDI-IHC, AmberGen, Inc.), GeoMx DSP® (NanoString, Inc.), and Imaging Mass Cytometry (IMC, Standard BioTools Inc.) were used in series with MALDI-MSI of N-glycans and extracellular matrix peptides on formalin-fixed paraffin-embedded tissues. Single cell omics protocols were performed before and after MALDI-MSI. The data suggests that for each modality combination, there is an optimal order for performing both techniques on the same tissue section. An overall conclusion is that MALDI-MSI studies may be completed on the same tissue section as used for antibody-directed single cell modalities. This work increases access to combined cellular and extracellular information within the tissue microenvironment to enhance research on the pathological origins of disease.

**Graphical Abstract:**

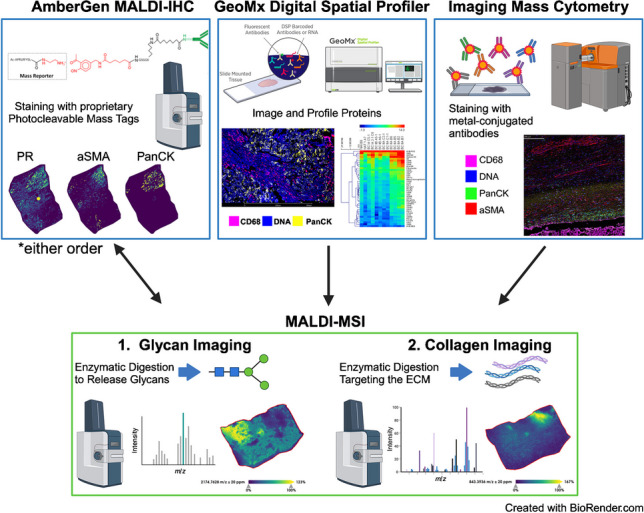

**Supplementary Information:**

The online version contains supplementary material available at 10.1007/s00216-023-04983-2.

## Introduction

The drive to understand the complexities of the tissue microenvironment has led to a plethora of multiplexed and multimodal spatial techniques [1–3]. Spatial omics has produced platforms that allow serial analyses of transcription or protein expression of single cells *in situ* through technologies such as RNA sequencing, immunohistochemistry, and immunofluorescence. Multimodal techniques have emerged that combine platforms to simultaneously evaluate very different aspects of the microenvironment, such as single cell expression paired with measurements of the physical properties of the tissue [4, 5]. The integration of mass spectrometry imaging with single cell spatial omics allows studies to synergistically add information from metabolomics, lipidomics, glycomics, or proteomics [6–11]. However, investigations covering the extracellular microenvironment simultaneous with cellular expression are limited.

The extracellular matrix (ECM) is a key regulator of the tissue microenvironment. The ECM is a proteinaceous composition of collagens and other glycoproteins that surrounds cells and provides control of cell-cell and cell-matrix signaling through diverse chemical composition arising largely from protein post-translational modifications. As such, localized ECM production is highly coordinated with enzyme transcription, protein synthesis, and metabolic pathways [12]. Primary post-translational modifications (PTMs) of the ECM include proline hydroxylation of collagen [13] and glycosylation that further fine-tune localized interactions within the tissue microenvironment. Collagen hydroxylation of prolines encodes collagen structure to expose or hide domains for protein and cell binding [14, 15]. Multiple types of cell surface glycosylation PTMs, including N-linked glycosylation, work to induce cell recruitment, alter protein-protein interactions, and produce gradient control between cells [16–18]. The differentiation, growth, homeostasis, and disease status of the tissue microenvironment are thus spatially controlled by both cellular and extracellular interactions that involve PTMs. The investigation of these interactions requires approaches that provide spatial information on cellular, ECM, and N-glycomic composition.

The majority of proteomic approaches targeting the extracellular matrix decellularize freshly obtained or fresh frozen tissue prior to enzymatic or chemical digestion [19–23]. There are very few ways to access the extracellular matrix in clinically archived formalin-fixed paraffin-embedded (FFPE) samples. A relatively recent approach to imaging the ECM in FFPE tissue uses collagenase type III sprayed onto tissue, which proteolytically targets the trypsin-resistant triple helical structure of collagens [24–26]. This allows spatial mapping of collagen structure, including proline hydroxylation, along with 40–60 other extracellular matrix proteins. After spraying with a chemical matrix, collagenase type III peptides are detected by matrix-assisted laser desorption/ionization mass spectrometry imaging (MALDI-MSI). Since collagenase digestion alters the tissue pathology, approaches have been developed that first apply hematoxylin and eosin (H&E) pathology staining for digital scanning followed by multiplexing with N-glycan imaging prior to collagenase digestion [27]. Completing H&E staining first also allows microscope measurements of collagen fibers by microscopy approaches such as second harmonic generation microscopy [28]. The removal of the N-glycans by peptide N-glycosidase digestion stereochemically increases access to the ECM protein structure [27]. After removal, subsequent imaging of the N-glycans is optional. Immunofluorescence [29] or immunohistochemistry [30] may also be done prior to collagenase digestion on the same tissue section, allowing reporting of cellular markers along with ECM distribution. Extracellular matrix imaging has advanced through the use of iterative washes and enzymatic digestions to report on the spatial distribution of multiple ECM components including N-glycans, elastin, and collagen within a single tissue section. This highly multiplexed approach, referred to as matrisome imaging, provides valuable insight into the spatial regulation of ECM components [31].

In this study, we evaluate the combination of single cell modalities with multiplexed N-glycan imaging followed by targeted ECM imaging by MALDI-MSI to investigate cellular and extracellular matrix composition from the same tissue section. Workflows using antibodies to target cellular markers included the Miralys™ photocleavable mass-tags (MALDI-IHC, AmberGen, Inc.), GeoMx® Digital Spatial Profiler (NanoString, Inc.), and Imaging Mass Cytometry (IMC, Standard BioTools Inc.) were evaluated in series with MALDI-MSI. These platforms are readily available commercially and in academic institutions yet require very specific protocols and proprietary materials for optimal performance. Each of these advanced platforms allows for increased potential for multiplexed analysis that includes the extracellular microenvironment when compared to classic immunohistochemical techniques. MALDI-IHC (AmberGen, Inc.) is an approach that uses mass-tagged antibodies to specific epitopes that are detected using mass spectrometry imaging to determine spatial distribution within a tissue. GeoMx Digital Spatial Profiler by NanoString uses affinity binding reagents targeting specific proteins that are linked to a DNA-sequence barcode by an ultraviolet (UV)-cleavable linker. The oligos are then cleaved by UV laser from a defined region of interest, collected, and counted [32]. Imaging mass cytometry uses rare metal-tagged antibodies to detect cellular features within a tissue. The metals are ablated with a laser and directed to a time-of-flight mass spectrometer for detection [33]. Here, the placement of multiplexed N-glycan MALDI-MSI and ECM MALDI-MSI was evaluated, either before or after each antibody-directed single cell workflow. This work broadens analytical access for researchers to investigate the combined extracellular microenvironment and cellular composition contributing to tissue pathology.

## Materials and methods

### Materials

2,5-Dihydroxybenzoic acid (DHB, 98.0%), 100% ethanol (pharmaceutical grade), ammonium bicarbonate for LC-MS (40867-50G-F), acetic acid glacial, bovine serum albumin, Advanced PAP Pen (5-mm tip width), 0.5 mL Ultrafree-MC centrifugal 0.45-µm filter devices, poly-l-lysine (P8920), IGEPAL CO-630 (542334), and octyl ß-d-glucopyranoside (OBG) (≥95% (HPLC), 50% (w/v) in H_2_O) were from Millipore Sigma (Burlington, MA/St. Louis, MO); Ted Pella StainTray™ (20 slide, base tray w/black lid), xylenes (semiconductor grade), water (LC-MS grade), and DyLight™ 650-4xPEG NHS ester were from Thermo Scientific (Waltham, MA); chloroform (HPLC Grade) was from Alfa Aesar (Haverhill, MA); isopropanol (Optima™ LC-MS Grade) was from Fisher Chemical™ (Hampton, NH); tris base (molecular biology grade) and tris-HCl (molecular biology grade) were from Promega (Madison, WI); and antigen retrieval buffer (100× Tris-EDTA buffer, pH 9.0) was from ABCAM (Cambridge, MA).

### Tissue specimens

For IMC experiments, liver and breast tissue sections were obtained through the MUSC tissue biorepository that banks tissue for all research and not only for the purposes of this study. The use of these tissues for the study was approved by MUSC IRB board as exemption #4. The FFPE breast cancer specimen used for MALDI-IHC was obtained from OriGene Technologies, Inc. (Rockville, MD) along with clinical annotations based on pathologist review of traditional immunohistochemistry (IHC) and hematoxylin/eosin (H&E) staining. This specimen was classified based on the provided annotations as adenocarcinoma of breast (ductal), TNM staging of pT2pN1apMX, minimum stage grouping IIB, 33% tumor, and PR+/ER+/HER2+. For GeoMx® studies, tissue was obtained through commercial vendors (TissueArray.Com LLC) with clinical characteristics of age, breast cancer stage, grade, and pathological diagnosis.

### MALDI-IHC

#### MALDI-IHC multiomic workflow

Methods were based on a prior work [34]. Briefly, tissue sections (3 µm, Zyagen, San Diego, CA) were mounted on conductive slides (Intellislides, Bruker Daltonics, Billerica, MA) which were pre-coated with poly-l-lysine by spreading 20 µL of 50% (v:v) poly-l-lysine containing 0.07% (v/v) of IGEPAL CO-630 detergent and drying the freshly coated slide using a laboratory oven at 80 °C. Tissue slides were processed in Coplin staining jars with 70 mL of fluid at room temperature unless otherwise specified. The following step was performed only in cases where direct glycan/ECM imaging was previously done—matrix was removed by washing for 5 min in excess methanol. The following steps were performed only in cases where direct glycan/ECM imaging was not previously done: FFPE tissue sections were pre-melted for 2 h in an oven at 60 °C. Tissue sections were then subjected to deparaffinization 3× with xylenes for 5 min each; and then 1× with 50% xylenes and 50% ethanol for 3 min. The following steps were performed in all cases and comprised rehydration, antigen retrieval, tissue blocking, and antibody incubation as follows (each treatment step in separate Coplin staining jars at room temperature unless otherwise specified): rehydration 2× with 100% ethanol for 2 min each, 1× with 95% ethanol for 3 min, 1× with 70% ethanol for 3 min, 1× with 50% ethanol for 3 min, and 1× with tris buffered saline (TBS; 50 mM Tris, pH 7.5, 200 mM NaCl) for 10 min. Antigen retrieval was achieved in 1× antigen retrieval buffer (100× Tris-EDTA buffer, pH 9.0) for 30 min at 95 °C and followed by cooling in the same Coplin staining jar for 30 min at room temperature. The tissue sections were encircled using the Advanced Hydrophobic (A-PAP) Barrier Pen (Sigma-Aldrich) and then blocked for 1 h with tissue blocking buffer (2% [v/v] normal serum [mouse 015-222-001 and rabbit 011-000-001, Jackson ImmunoResearch Laboratories, Inc. West Grove, PA] and 5% [w/v] BSA in TBS-OBG [TBS with 0.05% w/v OBG]). For staining with Miralys™ photocleavable mass-tag (PC-MT) antibodies (AmberGen, Billerica, MA), one tissue section on the slide was treated at 4 °C overnight with 200 µL of a solution containing 3.75 µg/mL of each antibody (Table S1) diluted in tissue blocking buffer and an adjacent (serial) tissue section on the same slide with 200 µL of a solution containing 3.75 µg/mL of a PC-MT isotype control IgG (see Table S1). Incubation was performed protected from light, in a humidified Ted Pella StainTray™ to avoid evaporation and with each tissue section surrounded using a hydrophobic Advanced PAP Pen (5-mm tip width) validated to eliminate contaminant detection by mass spectrometry and to facilitate fluid retention and low volume incubations. The slides were then washed 3× 5 min each with TBS followed by 3× 2 min each with 50 mM LC-MS grade ammonium bicarbonate (note, all solutions were in mass spectrometry grade water and all washes were performed using excess solution with the slides placed horizontally in a petri dish with gentle shaking). Tissue slides were ultimately dried for 1.5 h in a vacuum desiccation chamber. The dried tissue slides were illuminated for 10 min with 3 mW/cm^2^ of 365-nm light (UV Illumination Box, AmberGen Inc., Billerica, MA). DHB matrix was sublimated (Chemglass Life Sciences, Vineland, NJ) at 140 °C for 6 min and recrystallized at 55 °C with 1.0 mL 5% isopropyl alcohol in LC-MS water for 1–2 min in a petri dish with a filter paper.

#### MALDI-IHC instrumental parameters

The MALDI-IHC measurements were performed with a timsTOF fleX MALDI-2 instrument (Bruker Daltonics, Billerica, MA) using the following parameters: reflector mode positive ion mode for photocleavable mass tags (PC-MTs), transfer time set to 110 µs and pre-pulse storage at 15 µs. A laser spot size of 16 µm with beam scan resulting 20-µm spot size was used with a continuous raster beam scan, 300 laser shots/pixel, and 75% typical laser power setting. Image and spectral analyses normalized to total ion count (TIC) were performed using SCiLS Lab software (Bruker Daltonics, Billerica, MA).

### GeoMx® by NanoString

Human Breast TMA slide (TissueArray.Com LLC) was incubated for 4 h at 70 °C, deparaffinized, and rehydrated according to manufacturer’s specifications. Antigen retrieval was performed by heating the tissue to 95 °C for 15 min in Tris-EDTA, pH 9 and cooled for 30 min. The TMA slide was then washed and blocked per manufacturer’s specifications. Morphology markers (anti-CD68-Alex 647, Santa Cruz 1:50 dilution; anti-keratin-Alexa 594, Novus Biologicals, 1:25 dilution) along with oligo-conjugated antibodies from the Human Immune Cell Profiling core, Immune Cell Typing, and Immune Activation Status modules were diluted and incubated in blocking buffer W for 18 h at 4 °C in a humidified chamber. After incubation with morphology markers and profiling antibodies, ROIs can be chosen for subsequent experimental steps. ROIs are subject to UV light, which cleaves the oligos, based on properties of the areas of illumination (AOIs) that are designated during experimental design. In this case, each ROI was segmented into two AOIs, the first (segment 1) being defined as PanCK-positive and CD68-negative and the second (segment 2) having the opposite characteristics, PanCK-negative and CD68-positive. For both segments, there must also be positive DNA marker to ensure cell profiling. In this experiment, areas of equal size (600-µm diameter) were selected from twelve cores to profile the segmented cells. The GeoMx DSP technology has the ability to profile down to a single cell, but the cell segmentation strategy allows for the characterization of a single cell type within the tissue. Oligos were collected using microfluidics technology within the GeoMx instrument, counted on a NanoString nCounter MAX system, and analyzed using the Digital Spatial Profiling v2.0 software.

### Imaging mass cytometry (IMC)

Prior to acquisition, IMC was optimized for dilution of antibodies (Table S2), iridium-intercalator solution, and image acquisition strategies. FFPE breast and liver tissue sections at 5-µm thickness were heated for 1 h at 60 °C and wax removed using the following solutions: xylenes 2×3 min, 100% ethanol 1×1 min, Carnoy’s solution 2× 3 min, 100% ethanol 1×1 min, 95% ethanol 1×1 min, 70% ethanol 1×1 min, and HPLC grade water 2×3 min. Next, heat-induced epitope retrieval (HIER) was performed using Tris-EDTA pH9 HIER Buffer (Abcam ab93684), incubating for 20 min at 95 °C in the Decloaking Chamber™ NxGen (BioCare Medical). Tissue was encircled using the ImmEdge® Hydrophobic Barrier PAP Pen (Vector Laboratories) and nonspecific blocking performed using TBS/0.1% Triton X-100/3% BSA at room temperature in a humidity chamber for 1 h. Antibody master mix was prepared by diluting antibodies to experimentally determined concentrations in TBS/0.1% Triton X-100/1% BSA. Following the 1-h blocking incubation, blocking buffer was gently removed and antibody master mix was added to the tissue. Overnight incubation in a humidity chamber at 4 °C was followed the next day by washing slides in Coplin jars for 5 min: twice with PBS/0.2% Triton X-100 then 2× with PBS. After washing, Intercalator-Ir (Standard BioTools) was diluted 1:1000 in PBS and then incubated on tissue at room temperature in a humidity chamber for 30 min. The slide was washed 2× in HPLC water for 5 min then dried for at least 1 h in a vacuum desiccator.

Approximately 1000 µm × 1000 µm square regions were analyzed using the Standard BioTools Helios and Hyperion Tissue Imager. The instrument was tuned and calibrated using the 3-Element Full Coverage Tuning Slide (Standard BioTools) to ensure 175Lu counts exceed 750. Data was acquired at 1-µm step size, 200-Hz laser frequency, and energy of 1 or 2.5 dBa. ROIs were selected based on tissue histology and visualization of images was conducted using MCD Viewer v1.0.560.6 (Standard BioTools).

### MALDI-TOF mass spectrometry imaging

Each set of samples was prepared at the same time by enzymatic digestion, matrix spraying, and mass spectrometry imaging data collection to minimize potential batch effects. For samples preprocessed by IMC, MALDI-IHC, or by GeoMx®, samples proceeded directly to antigen retrieval to expose sites for enzyme digestion following standardized workflow protocols [24, 35–37].

#### Sample preparation for N-glycan imaging

Respective slides were heated for 1 h at 60 °C followed by deparaffinization and washing with the following solutions: xylenes 2×3 min, 100% ethanol 1×1 min, Carnoy’s solution 2× 3min, 100% ethanol 1×1 min, 95% ethanol 1×1 min, 70% ethanol 1×1 min, and HPLC grade water 2×3 min. The antigen retrieval process was performed using 10 mM citraconic anhydride buffer (pH 3) for 25 min in a vegetable steamer (approx. 95°C). PNGase F (PNGase F Prime, N-zyme Scientifics) was sprayed for 15 passes with an automated sprayer (M5, HTX Technologies) with the following parameters: 45 °C, 10 psi, 25 μL/min, 1200 velocity, nozzle at 40 mm above the tissue, and a 2.5-mm offset. The tissue was enzymatically digested in a preheated humidity chamber for 2 h at 37.5 °C. After digestion, CHCA matrix was prepared at 7 mg/mL in 50% acetonitrile and 0.1% TFA and sprayed by an automated sprayer (M5, HTX Technologies) for 10 passes with the following parameters: 79 °C, 10 psi, 70 μL/min, 1300 velocity, nozzle at 40 mm above the tissue, and a 3.0-mm offset.

#### Sample preparation for extracellular matrix imaging

After MALDI-MSI for N-glycans, the tissue sections were prepared for collagenase type III digestion targeting collagens and other extracellular matrix proteins. First, the N-glycans were washed from the tissue with the following solutions following previously reported protocols for N-glycan imaging and peptide imaging done in series [37]. Tissue was incubated 1 min each with 100% ethanol, 95% ethanol, 70% ethanol, water, high pH buffer (10 mM tris buffer, pH 9), water, low pH buffer (10 mM citraconic anhydride buffer, pH 3), and rinsed with water. HIER was done using 10 mM tris buffer (pH 9) for 25 min in a vegetable steamer (95 °C). Collagenase type III enzyme was prepared (0.1 µg/µL) in 10 mM ammonium bicarbonate with 1 mM CaCl_2_ and sprayed by an automated sprayer (M5, HTX Technologies) for 15 passes with the following parameters: 45 °C, 10 psi, 25 μL/min, 1200 velocity, nozzle at 40 mm above the tissue, and a 2.5-mm offset. The tissue was digested in a pre-heated humidity chamber at 37.5 °C for 5 h. CHCA matrix was prepared as 7 mg/mL in 50% acetonitrile and 1% TFA and sprayed using the same parameters described above. Once the matrix had dried, the slides were dipped in cold 5 mM ammonium phosphate and monobasic for 1 s and then dried in a desiccator.

#### MALDI-TOF mass spectrometry imaging

The data for the tissues was collected using a MALDI-QTOF (tims-TOF-fleX, Bruker) in positive ion mode sampling using 300 laser shots per pixel with laser power at 35%. Laser spot size was adjusted as needed depending on step sizes, which are reported for each tissue in the figure legends. N-Glycan analysis used m/z range 700–4000 with transfer time set to 90 µs and pre-pulse storage at 30 µs. Peptide analysis used m/z range 600–2500 with transfer time set to 80 µs and pre-pulse storage at 20 µs. MALDI-MSI data was imported into SCiLS Lab 2022 and normalized to total ion current (TIC). Maximum peak intensities were extracted from SCiLS Lab and evaluated using Excel and GraphPad (Prism v9). Difference in peak intensity for each modality was calculated as 100−((MALDI MSI First/Modality First)*100). Spectra of MALDI-MSI first vs other imaging modality first were compared using mMass [38].

## Results and discussion

### Overview

Single cell modalities work to map single cell expression across tissue sections but have some limitations in reporting other aspects of the tissue microenvironment such as the extracellular matrix. In the current study, we investigated strategies for pairing antibody-based single cell technology with MALDI-MSI of N-glycans and extracellular matrix proteins on the same tissue section. Standardized protocols for each workflow were used to ensure optimal performance (Table 1). MALDI-MSI of both N-glycans and extracellular matrix were completed either before or after the single cell imaging modality, comparing for peak intensity variation and introduction of imaging artifacts in the MSI data. Workflows were purposefully utilized directly after each other in series towards determining best placement of each workflow, either before or after MALDI-MSI. The data suggests that for each combination, there is an optimal order for performing the single cell modality and MALDI-MSI on the same tissue section. An overall goal is to develop spatial multiomic strategies that allow researchers to investigate both cellular and extracellular features within the tissue microenvironment.

**Table 1:** Comparison of method parameters for each workflow. Workflows were done in sequence using manufacturer’s protocol method parameters. The dewaxing procedures were the same and are described in the “Materials and methods” section. Separate antigen retrieval was performed for MALDI-MSI of N-glycans and ECM peptides to expose the correct sites for protease access

	Initial heating time (h)	Initial heating temperature	Antigen buffer (pH)	Antigen retrieval device	Antigen retrieval temperature	Antigen retrieval time (min)	Type of antibody
MALDI-MSI: N-Glycans	1	60 °C	Citraconic, 10 mM pH 3.0 *prepared in house*	Vegetable steamer	95 °C	25**	NA
MALDI-MSI: ECM peptides	1	60 °C	Tris, 10 mM pH 9.0 *prepared in house*	Vegetable steamer	95 °C	25**	NA
AmberGen MALDI HiPLEX-IHC	2	60 °C	Tris-EDTA, pH 9.0 *commercial preparation*	Water bath	95 °C	30	Photocleavable mass-tags
GeoMx Digital Spatial Profiler	4	70 °C	Tris-EDTA, pH 9.0 *commercial preparation*	Rice cooker	95 °C	15	Fluorescent and oligo-conjugated
Imaging Mass Cytometry (IMC)	1	60 °C	Tris-EDTA, pH 9.0 *commercial preparation*	Decloaker	95 °C*	20	Metal-conjugated

^**^Time was reduced to 15 min for the breast TMA to avoid tissue loss

### MALDI-MSI of N-glycans and ECM imaging in combination with MALDI-IHC

**Fig. 1:**
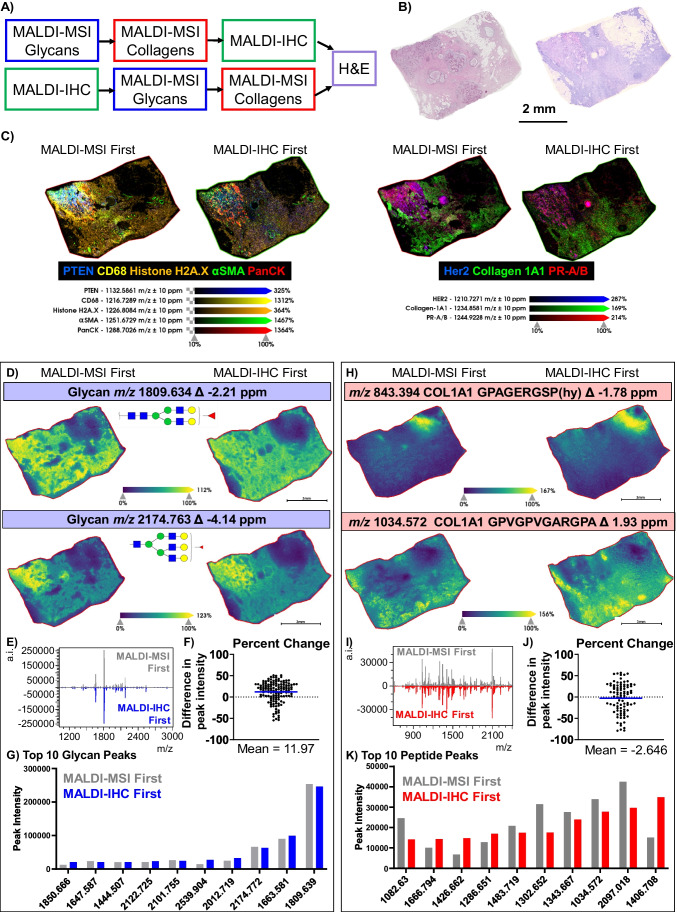
Combination of MALDI-MSI and MALDI-IHC on Human Breast Cancer Tissue. **A** An overview of the workflows performed. **B** Example H&E staining of two separate sections of the breast tissue; before workflow is started (left) and after workflows are completed (right). **C** Two sets of AmberGen MALDI-IHC images from slides processed by MALDI-MSI first (left tissue) and IMC first (right tissue). Markers visualized and their mass tags are labeled below images. MALDI-IHC step size was 20 µm. **D** Comparisons of two N-glycan ion images (1809.643 and 2174.763 m/z, 40 µm) from MALDI-MSI first and MALDI-IHC first along with (**E**) the whole spectra of N-glycans, (**F**) mean percent change when compared to MALDI-MSI first, and (**G**) the 10 most intense N-glycan peaks, ordered by MALDI-IHC first. **H** Images of collagen peptides with masses 843.394 m/z and 1034.572 m/z are compared for MALDI-MSI first and MALDI-IHC first. Step size of 40 µm was used. **I** The spectra of extracellular molecules are compared. **J** The percent difference in peak intensity displayed for a subset of selected peaks with respect to MALDI-MSI first. **K** Top 10 collagen peaks of highest intensity in MALDI-IHC first are compared

MALDI-IHC uses novel photocleavable mass-tags (PC-MTs) conjugated to antibody probes as reporters to detect specific markers and cell types in tissues [34]. Detection is done by MALDI-MSI, allowing highly multiplexed imaging of antibodies tagged with PC-MTs. MALDI-IHC has been used as a targeted approach to guide laser capture microdissection followed by tryptic proteomics and paired with untargeted mass spectrometry imaging of tryptic peptides [39, 40]. In the present study, combinations of MALDI-IHC with N-glycan and ECM imaging by MALDI-MSI were explored on breast cancer tissue followed by hematoxylin and eosin staining (Fig. 1A and B). The panel of MALDI-IHC probes was targeted at 28 markers including PTEN, CD68, Histone H2AX, alpha-smooth muscle actin, pan cytokeratin, human epidermal growth factor receptor 2, collagen type I alpha 1, and progesterone receptor A/B (Supplemental Table 1). The combination of these techniques may have an impact on the integrity of the tissue and downstream pathology staining as the final tissue showed signs of laser ablation while using controlled laser parameters (Supplemental Figure 1). Combined ion images demonstrate detection of multiple PC-MTs either with MALDI-MSI completed before or after MALDI-IHC (Fig. 1C). Similar localization of the mass-tags was observed regardless of the order of placement for MALDI-MSI of N-glycans and collagens with respect to MALDI-IHC with certain antibodies showing some decreases in detection after MALDI-MSI was completed first (Supplemental Figure 2). However, additional work showed certain PC-MTs exhibited decreased intensity when imaged after MALDI-MSI (Supplemental Figure 3). We hypothesize that decreased detection after MALDI-MSI is due to decreased antibody binding as it is likely that certain epitopes are altered by the MALDI matrix.

MALDI-MSI produced similar ion images regardless of the order of completion. For N-glycan imaging, the spatial distribution of two N-glycans, *m/z* 1809 and 2174, are shown as examples (Fig. 1D, Supplemental Figure 4). When comparing intensities of 139 peaks, N-glycan peaks showed a slight increase when MALDI-IHC was completed first with a mean percent change of 12.0 with ± 24.3% variance over all peaks (Fig. 1E, F). The most abundant N-glycan peaks show almost identical peak intensities from both orders of workflow combinations (Fig. 1G). ECM imaging also demonstrates comparable spatial distributions of peptides and peak intensities (Fig. 1H and I, Supplemental Figure 5). Collagen intensities showed a mean percent change of −2.6 with ± 35.1% variance over all peaks when MALDI-IHC was performed first (Fig. 1J). However, certain peaks were almost twice as intense in MALDI-IHC first and others more intense in MALDI-MSI first (Fig. 1K). The difference in peak intensities is greater than section-to-section variability previously reported [41] and could be due to differential denaturing of collagen digestion sites dependent on the order of workflows. Completing MALDI-IHC prior to N-glycomic and ECM imaging allowed consistent antibody detection by PC-MT with a minimal alteration of N-glycan or collagen peptide peak intensities.

### MALDI-MSI of N-glycans and ECM imaging with NanoString GeoMx® digital spatial profiler

**Fig. 2:**
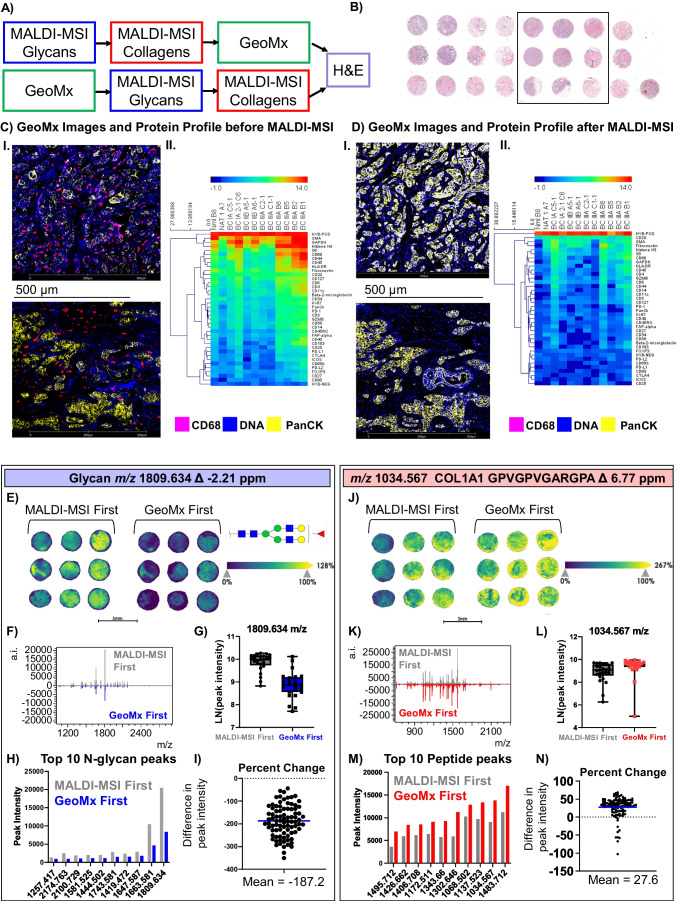
Combination of MALDI-MSI and GeoMx on human breast tissue microarray. **A** An overview of the workflows performed. **B** Representative H&E of the breast TMA. Boxed region signifies cores shown in **E** and **J** (**C**) **I** Two areas of illumination from two TMA cores imaged by GeoMx before MALDI-MSI and **II** the associated protein profiles from the 12 areas illuminated within the TMA. (**D**) **I** The same cores and areas of illumination are shown for the slide processed by GeoMx after MALDI-MSI and **II** the associated protein profiles displayed as a heatmap. In the fluorescence images, markers include DNA (blue), PanCK (yellow), and CD68 (pink) and the heatmaps profile cells segmented by positive PanCK status within an ROI. **E** Comparison of N-glycan ion images (1809.634 m/z) taken with 30-µm step size along with (**F**) the whole spectra of N-glycans and (**G**) quantitation of the 1809 m/z peak intensities visualized by individual patient. **H** The 10 most intense N-glycan peaks over the whole TMA are shown, ordered by GeoMx first. **I** The differences in peak intensities are highlighted by the percent change when comparing GeoMx first to MALDI-MSI first. **J** Peptide images for m/z 1034.572 with step size of 30 µm are compared for MALDI-MSI first and GeoMx first. **K** The spectra of extracellular molecules are compared and **L** peak intensities for m/z 1034.572 are visualized by individual patient. **M** The top 10 peaks of highest intensity in GeoMx first are compared.** N** The percent difference in peak intensity for a subset of selected peaks with respect to MALDI-MSI first shows that on average GeoMx first peaks had higher intensities than MALDI-MSI first

The GeoMx Digital Spatial Profiler (DSP) allows for high-resolution imaging by detection of fluorescent probes and multiplexed detection of proteomic data using antibodies coupled to photocleavable oligonucleotide tags. A human breast tissue microarray was used for exploration of the multimodal techniques as outlined in Fig. 2A followed by H&E staining (Fig. 2B). The TMA was comprised of duplicates of breast cancer and normal adjacent tissue from six female patients and one male adrenal gland as a tissue control.

The GeoMx DSP workflow uses up to 4 fluorescent antibodies to stain the tissue for morphology markers to aid in selection of regions of interest (ROIs) for protein profiling. Regions of interest are then selected to profile multiple target proteins using a panel of oligo-conjugated antibodies. Here, each TMA was stained for DNA, pan cytokeratin, and CD68 (Fig. 2C and D (I)). Regions for protein profiling by photocleavable oligonucleotide tags by GeoMx DSP were selected from within these cores based on stained areas. While pan-cytokeratin staining appears robust after MALDI-MSI, detection of CD68 is decreased when MALDI-MSI was done prior to GeoMx (Fig. 2C and D). The heatmaps displayed in Fig. 2C and D (II) profile the proteins from PanCK-positive segmented cells from 12 cores because CD68-positive cells are less abundant when GeoMx is performed after MALDI-MSI. A decrease in detection of protein profiles by GeoMx was observed when MALDI-MSI of N-glycans and collagens was completed first (Fig. 2D), potentially due to denaturation of the antibody binding epitopes as found in MALDI-IHC.

When GeoMx is done first, the N-glycan MALDI-MSI data is strikingly different. The ion images and overall spectrum show decreased peak intensities (Fig. 2E and F). For example, an abundant N-glycan peak such as m/z 1809 showed over a 2-fold change (Fig. 2G). The decreases in N-glycan intensities are not only apparent in the most abundant peaks (Fig. 2H) but also when 86 peaks are compared in GeoMx first with respect to MALDI-MSI first, where a decrease in mean peak intensities of −187.2 with ± 68.5% variance was seen (Fig. 2I). This is the only combination of methods investigated that shows large differences in N-glycomic peak intensities (Supplemental Figure 6). This large decrease in N-glycan intensities was also demonstrated on additional tissues when comparing N-glycan signal following GeoMx (Supplemental Figure 7). We hypothesize that the differences observed for N-glycan spectra and images may be due to the longer initial incubation at higher temperatures during the GeoMx protocol prior to MALDI-MSI. A possible alternate workflow could place GeoMx between N-glycomic imaging and ECM imaging to avoid glycan signal differences.

MALDI-MSI data from ECM imaging produced similar spectra. Ion images from ECM peptides showed increased peak intensities when GeoMx was performed first compared to MALDI-MSI (Fig. 2J and K). An example is seen with m/z 1034.567 which increased by approximately 25% when compared with MALDI-MSI done first (Fig. 2L and M). When comparing peak intensities of 115 collagenase peaks, there was an increase in mean percent difference of 27.6 ± 28.4% when GeoMx was completed first (Fig. 2N, Supplemental Figure 8).

To summarize, protein profiling within the target area by GeoMx showed decreased protein expression counts when MALDI-MSI was completed first. Additionally, N-glycans showed decreased peak intensities but similar spatial localization when GeoMx was completed first while ECM peptides showed increased peak intensities. To obtain high-quality cell marker imaging, GeoMx is optimal when completed prior to MALDI-MSI with an acknowledged tradeoff in decreased N-glycan signal.

### MALDI-MSI in combination with imaging mass cytometry

Human breast cancer tissue sections were used to evaluate the combination of imaging mass cytometry (IMC) and MALDI-MSI. A panel of metal-conjugated antibodies had already been developed and optimized for IMC and a selection of these antibodies were used in this work (Supplemental Table 2). A serial section of the breast tissue was subject to IMC antibody staining and imaging followed by MALDI-MSI using multiplexed enzymatic digestion. The same methods were completed on an adjacent section from the same breast tissue in reverse order, with MALDI-MSI completed prior to IMC, to evaluate which sequence of method steps yielded higher quality data. A key difference in IMC compared to other single cell modalities is that IMC studies are typically focused on very specific histological features within small regions (500 × 500 µm). However, processing of the tissue to apply the metal-conjugated antibodies occurs over the entire tissue section and MALDI-MSI was performed over the entire tissue section.

**Fig. 3:**
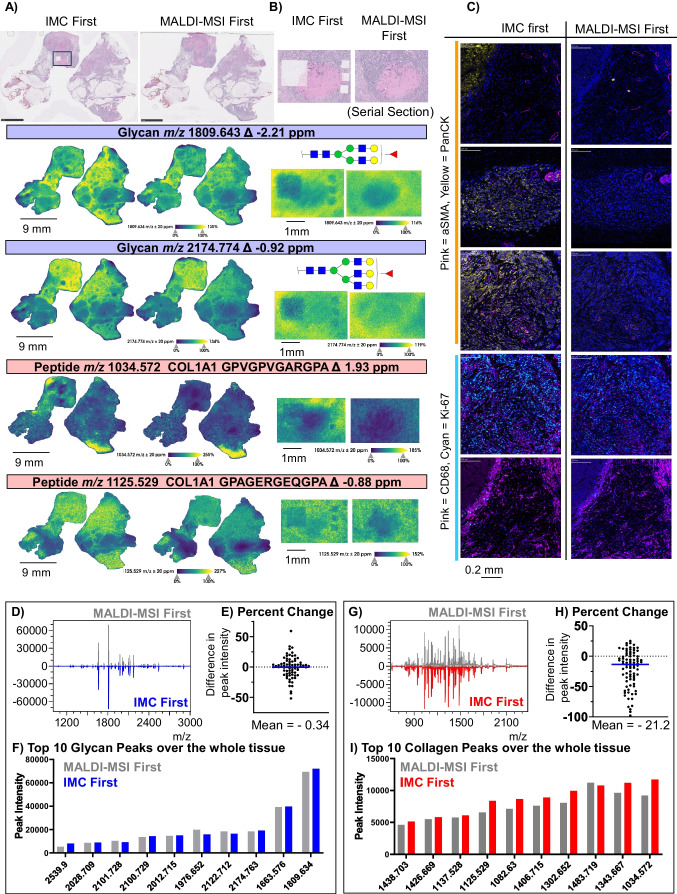
Combination of MALDI-MSI and IMC on human breast cancer tissue. **A** Whole tissue H&E and MALDI-MSI images of breast samples processed by MALDI-MSI first (left column) and IMC first (right column) both taken with 80-µm step size. Images from two N-glycan structures (1809.625 and 2174.763 m/z) are shown followed by two extracellular matrix molecules (1034.572 m/z and 1125.529 m/z). **B** Magnified H&E and higher resolution (step size of 20 µm) MALDI-MSI images of area analyzed by IMC that shows the spot of tissue ablation from the IMC laser for matched N-glycan/peptide structures from **A**.** C** IMC images from slides processed by IMC first (left column) and MALDI-MSI first (right column). Markers visualized are labeled by row. **D** Comparison of N-glycan spectra showing MALDI-MSI first on top and IMC first flipped on the bottom. **E** Differences in peak intensity relative to MALDI-MSI first. **F** Top 10 peaks of highest intensity in IMC first are compared for N-glycans. **G** Comparisons of the whole spectra collagens. **H** The percent difference in peak intensity for a subset of selected peaks with respect to MALDI-MSI first. **I** Ten highest intensity peaks ranked by IMC first compared to intensities of MALDI-MSI first. **(D–I** are measured from the whole tissue)

A noticeable difference was present for both N-glycans and collagen peptides in the tissue that had been processed by IMC first, but the spatial distribution of surrounding tissue is similar when comparing both series (Fig. 3A). Comparison of high spatial resolution mass spectrometry images of the IMC analyzed site (annotated in black outline, top left Fig. 3A) demonstrated that performing IMC first almost completely ablated the tissue and may contribute to differences seen in peak intensity within IMC-ablated regions (Fig. 3A, B). Over the entire tissue, N-glycan spectra were very similar and when comparing peak intensities of 77 N-glycan peaks, minimal differences were observed when IMC was completed first (mean of −0.34 ± 17.8%) (Fig. 3D–F and Supplemental Figure 9). For collagen peptides, a mean difference in peak intensity of −21.2 with ± 30.0% variance was observed over 82 selected peaks (Fig. 3G–I and Supplemental Figure 10). While the overall spectra for N-glycans and collagens are generally similar regardless of the order of methods, completing IMC first resulted in the ablation region showing differential intensity by MALDI-MSI. Variable intensities were observed in the small regions by MALDI-MSI approaches for N-glycans and ECM peptides when compared to the MALDI-MSI control tissue. The site of IMC ablation is distinct in the H&E stains that were performed after investigated techniques (Fig. 3A, B). The lower signal intensities are likely due to almost total tissue ablation by the IMC laser. The regions of IMC ablation must be annotated to avoid attributing differences in signal to pathology in a tissue of interest.

When MALDI-MSI was completed prior to IMC, antibody staining was diminished, following similar patterns seen by MALDI-IHC and GeoMx (Fig. 3C). In IMC processes, it was observed that the counterstain for DNA was not decreased. The counterstain is a cationic nucleic acid intercalator that is incubated on the tissue in a separate step after the metal-conjugated antibodies so as not to disrupt the binding of antibodies to their epitopes. The similar staining of DNA is expected as there is no epitope recognition involved and thus detection would not be susceptible to denaturation of structure. Imaging mass cytometry before and after MALDI-MSI was replicated on liver cancer tissue sections and similar results were observed (Supplemental Figures 11–13). In summary, images with similar peak intensities and similar image distribution were observed when IMC was performed first as compared to MALDI-MSI first. Although the whole tissue MALDI-MSI images are similar, the ablation region should be annotated in analyses.

## Conclusions

**Fig. 4:**
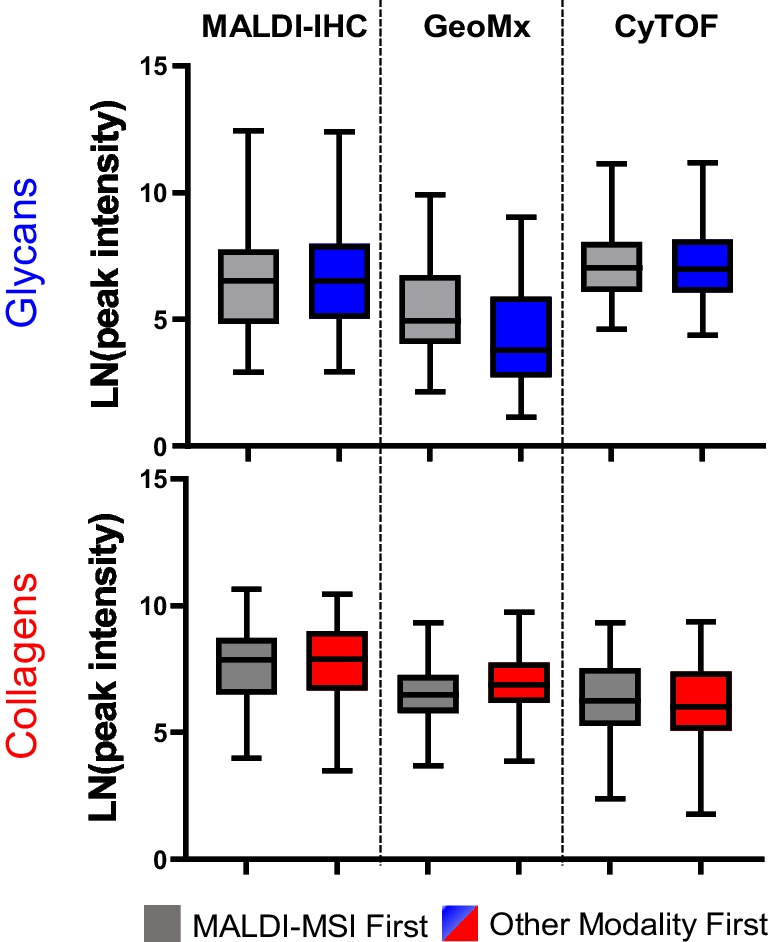
Summary of MALDI-MSI peak intensities of N-glycans and collagen peptides with MALDI-MSI first or the other single cell imaging modality first

The combination of antibody-directed single cell spatial ‘omic workflows with MALDI-MSI allows researchers to probe both cellular and extracellular interactions from a single tissue section when investigating biological hypotheses. One important consideration when deciding which antibody-directed spatial technique to use is the size of the region of interest being investigated. MALDI-IHC is more beneficial for studies requiring a scan of the whole tissue down to cellular resolution [34, 40] while IMC and GeoMx work on smaller, target regions of interest within the tissue. This wide field capability can be an important advantage for identifying smaller ROIs, especially in conjugation with higher spatial resolution techniques such as IMC. While all methods explored add complementary spatially resolved data, there were limitations to combining the workflows. Certain antibody staining showed diminished detection for certain antibodies when performed after MALDI-MSI. It is possible that this could be due to the drying of the acidic MALDI matrix, thereby disrupting specific epitopes and thus accounting for certain antibodies that performed poorly after MALDI-MSI. This was observed using PC-MTs in the MALDI-IHC approach and demonstrated that certain antibodies are detected equally before or after MALDI-MSI while others are decreased after MALDI-MSI. This study used alpha-cyano-4-hydroxycinnamic acid (CHCA) matrix for detection of N-glycans and collagen peptides as previously reported in protocols. Other matrices should be explored for potential effects in combination with antibody-directed single cell imaging. The GeoMx protocol appeared to result in a decrease in N-glycan signal which was attributed to increased heating time; this is a future point of evaluation. An additional finding was that different tissues performed similarly for specific workflows when evaluating the order placement of MALDI-MSI, suggesting that this evaluation approach may be useful in diverse tissues. Figure 4 summarizes comparisons of the workflow placement of MALDI-MSI of N-glycans and collagens with antibody-directed single cell imaging. An overall conclusion is that single cell spatial omics using antibody detection can be optimally performed in tandem with MALDI-MSI on the same tissue section once the order of the complementary techniques is determined.

### Supplementary Information

Below is the link to the electronic supplementary material.

Supplementary file1 (DOCX 10769 KB)

## Declarations

### Source of biological material

Tissues from MUSC’s Biorepository & Tissue Analysis shared resource were approved for use for this study by MUSC IRB board as exemption #4.
