# Supplementary material for: Evaluation of antibody-based single cell type imaging techniques coupled to multiplexed imaging of N-glycans and collagen peptides by matrix-assisted laser desorption/ionization mass spectrometry imaging
Source: Anal Bioanal Chem. 2023 Oct 16;415(28):7011–24. doi: 10.1007/s00216-023-04983-2 (PMC10632234; doi:10.1007/s00216-023-04983-2)
Supplement: Supplementary file 1 — Supplementary file1 (DOCX 10769 KB) [file 216_2023_4983_MOESM1_ESM.docx]

Supplementary Information

Evaluation of Antibody-Based Single Cell Type Imaging Techniques Coupled to Multiplexed Imaging of N-Glycans and Collagen Peptides by Matrix-assisted Laser Desorption/Ionization Mass Spectrometry Imaging

**Jaclyn Dunne^1^, Jake Griner^1^, Martin Romeo^2^, Jade Macdonald^1^, Carsten Krieg^3^, Mark Lim^4^, Gargey Yagnik^4^, Kenneth J. Rothschild^4,5^ Richard R. Drake^1^, Anand S. Mehta^1^, *Peggi M. Angel^1^**

1. Department of Cell and Molecular Pharmacology & Experimental Therapeutics, Medical University of South Carolina, Charleston, SC 29425 USA
2. Translational Science Laboratory, Hollings Cancer Center, Charleston, SC 29425 USA
3. Department of Pathology and Laboratory Medicine, Medical University of South Carolina, Charleston, SC 29425 USA
4. AmberGen, Inc., 44 Manning Road, Billerica, Massachusetts 01821, USA
5. Boston University, Department of Physics and Photonics Center, Boston, MA 02215 USA

Supplementary Tables

**Supplemental Table 1. AmberGen Antibody Probes.** All antibodies used in the MALDI-IHC workflow are monoclonal antibodies (clone numbers provided in table). Photocleavable peptide based mass-tags (PC-MTs) are produced using an amine-terminal fluorenylmethoxycarbonyl (Fmoc)-protected photocleavable linker in Fmoc-based solid-phase peptide synthesis. The PC-MTs are then conjugated directly to antibodies to make the Miralys™ Probes. The table below includes the target of the antibody, the m/z of the associated PC-MT reporter ion, and AmberGen catalog number. All Probes were used at a 3.75 µg/mL staining concentration.

| **Catalog #** | **Target** | **Clone #** | **Host** | **Species Reactivity** | **PC-MT-(ID)** | **PC-MT Reporter Ion (M+H)+** |
| --- | --- | --- | --- | --- | --- | --- |
| AP1001172 | Actin-αSM (α-Smooth Muscle Actin) | D4K9N | Rabbit | M,Rat,H | -2.01 | 1,251.68 |
| AP100153 | Collagen-1A1 (COL1A1) | E8F4L | Rabbit | M,H | -Iso1.06 | 1,234.87 |
| AP1001171 | FN1 (Fibronectin) | E5H6X | Rabbit | H | -12.03 | 1,068.60 |
| AP1001170 | Histone H2A.X | D17A3 | Rabbit | M,Rat,H,Mnky | -Iso1.04 | 1,226.82 |
| AP1001169 | Na/K ATPase-α1 | D4Y7E | Rabbit | H | -Iso1.03 | 1,222.79 |
| AP1001174 | PanCK (Pan-Cytokeratin) | C11 | Mouse | M,Rat,H,Mnky | -7.13 | 1,288.72 |
| AP1001122 | VIM (Vimentin) | D21H3 | Rabbit | M,Rat,H,Mnky | -Iso1.05 | 1,230.84 |
| AP1001187 | CD11b | D6X1N | Rabbit | H | -1.08 | 1,467.81 |
| AP1001186 | CD20 | E7B7T | Rabbit | H | -15.00 | 997.53 |
| AP1001181 | CD3ε | D7A6E | Rabbit | H | -10.07 | 1,161.65 |
| AP100173 | CD4 | EPR6855 | Rabbit | H | -1.02 | 1,293.75 |
| AP1001179 | CD44 | E7K2Y | Rabbit | M,Rat,H | -14.03 | 1,102.59 |
| AP1001124 | CD68 | D4B9C | Rabbit | H | -Iso1.02 | 1,216.75 |
| AP100152 | CD8α | D8A8Y | Rabbit | H | -1.04 | 1,350.76 |
| AP1001183 | ECAD (E-Cadherin/CDH1) | 4A2 | Mouse | M,Rat,H | -10.00 | 930.56 |
| AP1001193 | FoxP3 | D2W8E | Rabbit | H | -1.09 | 1,494.82 |
| AP1001190 | GZMB (Granzyme B) | D6E9W | Rabbit | M,H | -11.00 | 938.53 |
| AP1001123 | HER2 | D8F12 | Rabbit | M,H | -Iso1.01 | 1,210.74 |
| AP1001182 | Histone H3 | D1H2 | Rabbit | M,Rat,H,Mnky | -1.15 | 1,782.94 |
| AP1001177 | HLA-G | E8N9C | Rabbit | H | -14.00 | 988.55 |
| AP1001184 | Ki67 | 8D5 | Mouse | H | -1.03 | 1,320.76 |
| AP1001114 | NCAM1 (CD56) | E7X9M | Rabbit | M,Rat,H | -13.00 | 970.52 |
| AP1001188 | PD1 (PDCD1) | D4W2J | Rabbit | H | -1.10 | 1,524.83 |
| AP1001194 | PDGFR-B (PDGF Receptor β) | 28E1 | Rabbit | M,Rat,H | -12.05 | 1,125.63 |
| AP100197 | PD-L1 (CD274) | E1L3N | Rabbit | H | -1.06 | 1,407.79 |
| AP1001196 | PDPN (Podoplanin) | LpMab-12 | Mouse | H | -12.00 | 954.56 |
| AP1001176 | PR-A/B (Progesterone Receptor A/B) | D8Q2J | Rabbit | H | -Iso1.08 | 1,244.93 |
| AP1001195 | PTEN | D4.3 | Rabbit | M,Rat,H,Mnky,Dg | -14.04 | 1,132.60 |

**Supplemental Table 2. CyTOF Antibodies.** All antibodies were sourced from Fluidigm (Standard BioTools), already metal-conjugated, or from other vendors and conjugated in-house. The metal associated with each marker is displayed in the table.

| **Isotope** | **Element** | **Marker** | **Clone** | **Vendor** |
| --- | --- | --- | --- | --- |
| 141 | Pr | aSMA | 1A4 | Fluidigm |
| 148 | Nd | Pan-keratin | C11 | Fluidigm |
| 159 | Tb | CD68 | KP1 | Fluidigm |
| 162 | Dy | CD8a | CD8/144B | Fluidigm |
| 168 | Er | Ki-67 | B56 | Fluidigm |
| 191/193 | Ir | Nucleic acid | N/A | Fluidigm |

Supplementary Figures

# AmberGen MALDI-IHC with MALDI-MSI

**
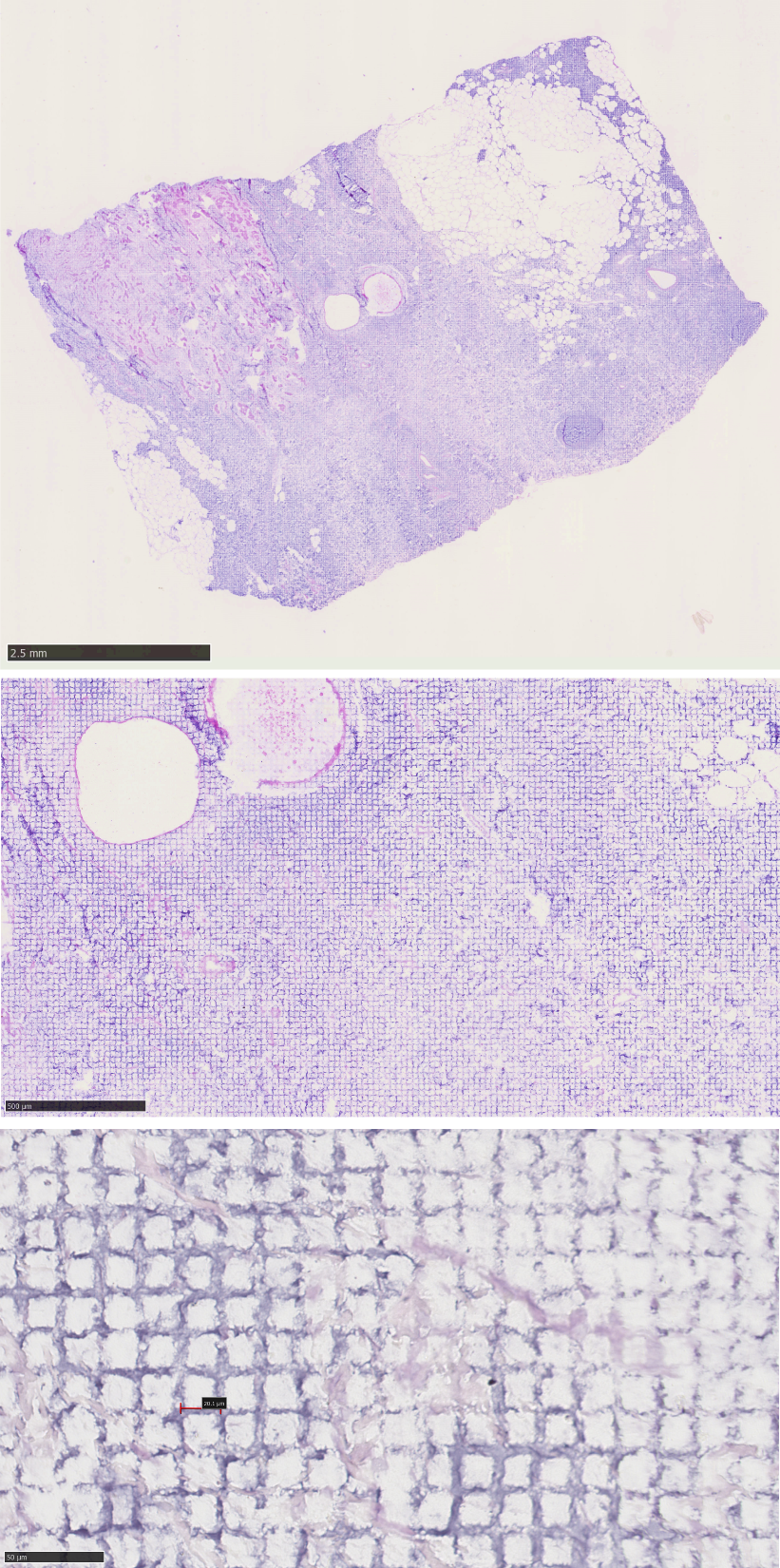
Supplemental Figure 1.** Example of laser ablation marks at a 20 µm step size, 35% laser power, 300 shots per pixel after completing a workflow of MALDI-IHC multiplexed with MALDI-MSI of N-glycans and MALDI-MSI of ECM followed by hematoxylin & eosin staining. Slides were scanned on a Hamamatsu Photonics NanoZoomer.

**
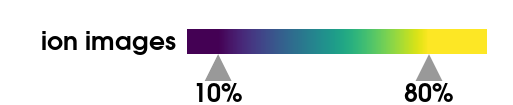
Supplemental Figure 2. Individual Images of AmberGen MALDI-IHC Markers.** Some differences are visually detected in the intensity patterns when either protocol was performed first (e.g., HER2, vimentin, CDH1). Images were normalized in the same dataset using total ion current.

3 mm

| **Marker** | **PR** | **His H2A.X** | **Her2** | **PTEN** | **CD68** |
| --- | --- | --- | --- | --- | --- |
| AmberGen MALDI-IHC First | 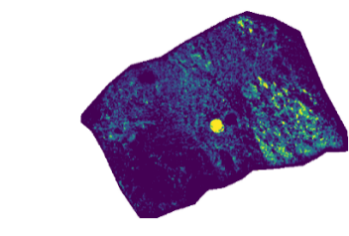 | 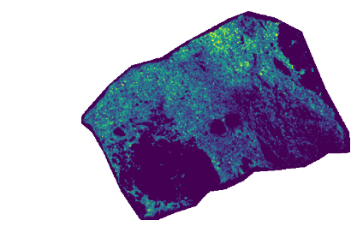 | 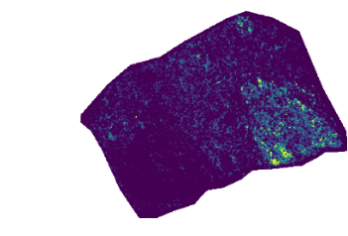 | 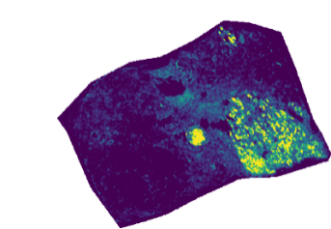 | 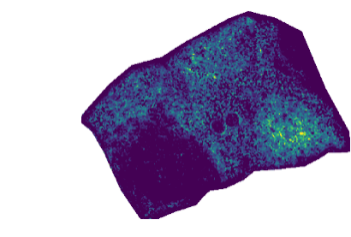 |
| MALDI-MSI First | 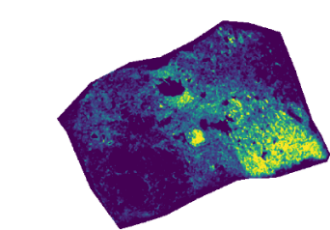 | 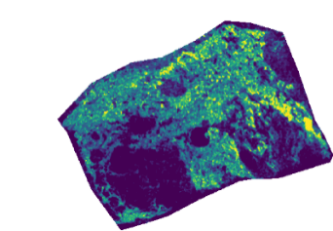 | 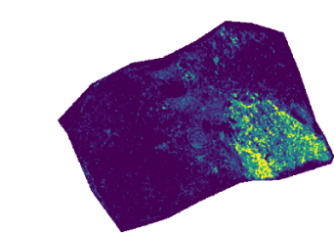 | 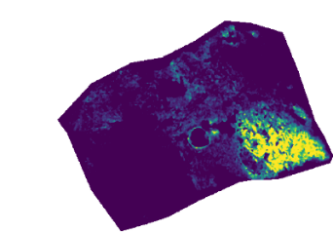 | 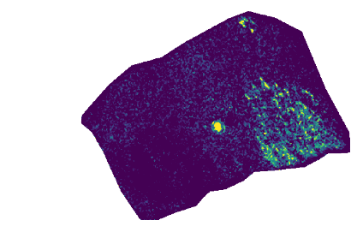 |
| **Marker** | **aSMA** | **PanCK** | **Vimentin** | **Collagen 1A1** | **CDH1** |
| AmberGen MALDI-IHC First | 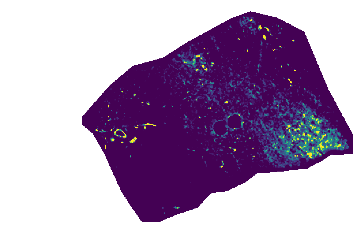 | 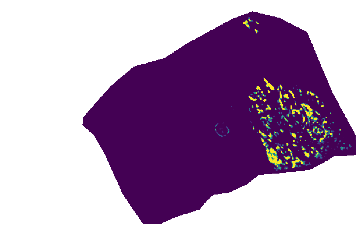 | 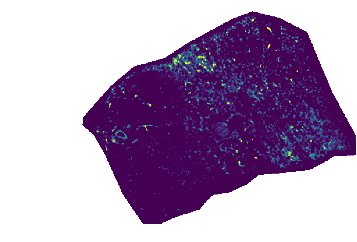 | 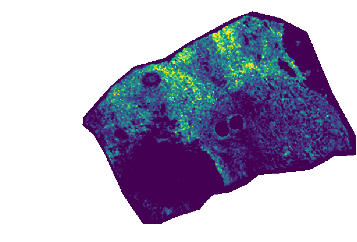 | 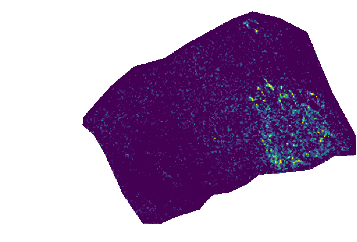 |
| MALDI-MSI First | 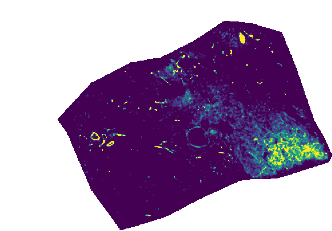 | 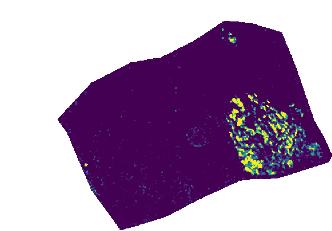 | 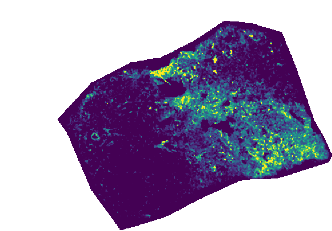 | 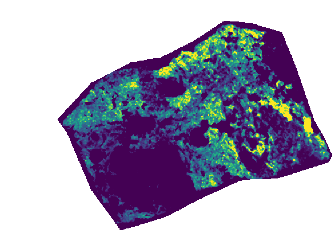 | 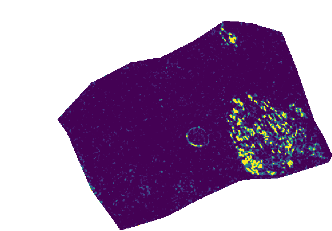 |


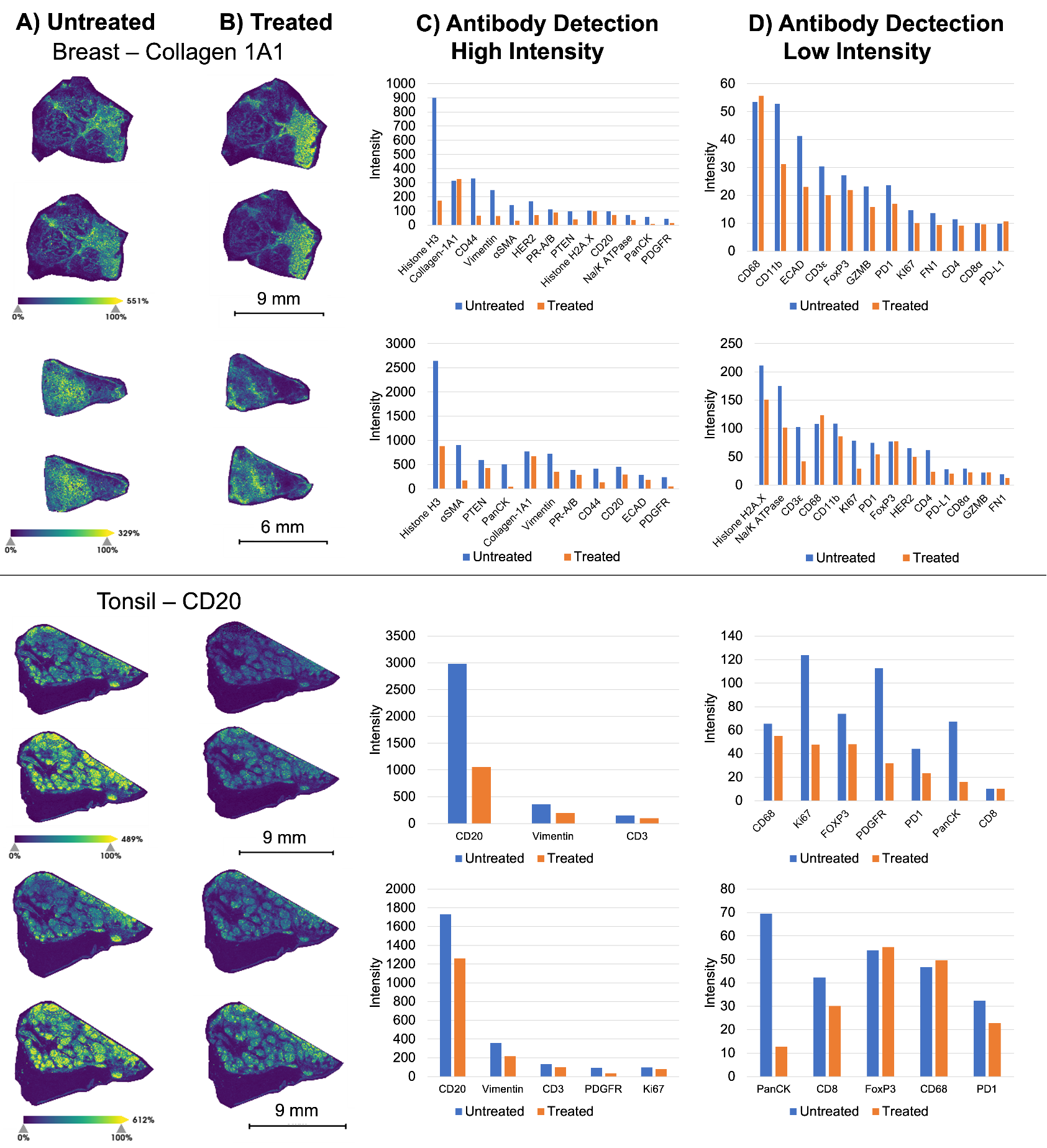
**Supplementary Figure 3. AmberGen MALDI-IHC was evaluated on additional breast (above horizontal line) and tonsil tissues (below line).** Untreated refers to a new FFPE tissue processed by the MALDI-IHC workflow. Treated tissue is FFPE tissue that has been digested by PNGase F Prime and Collagenase type III enzymes and processed by MALDI-MSI prior to MALDI-IHC. In A and B pairs of tissues stacked vertically are from adjacent tissue sections. Generally, intensities of antibody detection vary for given epitopes. We hypothesize that some epitopes may not be as sensitive to denaturation by the MALDI matrix and thus bind antibodies similarly when comparing before or after MALDI-MSI. C and D were calculated from the average signal of replicates.


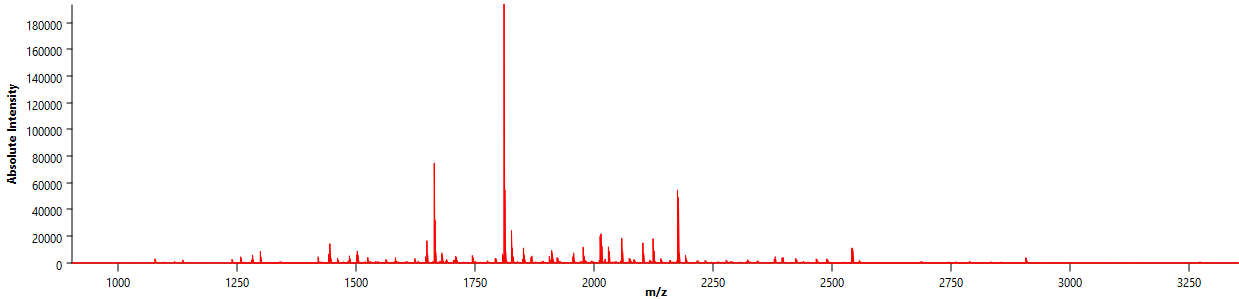

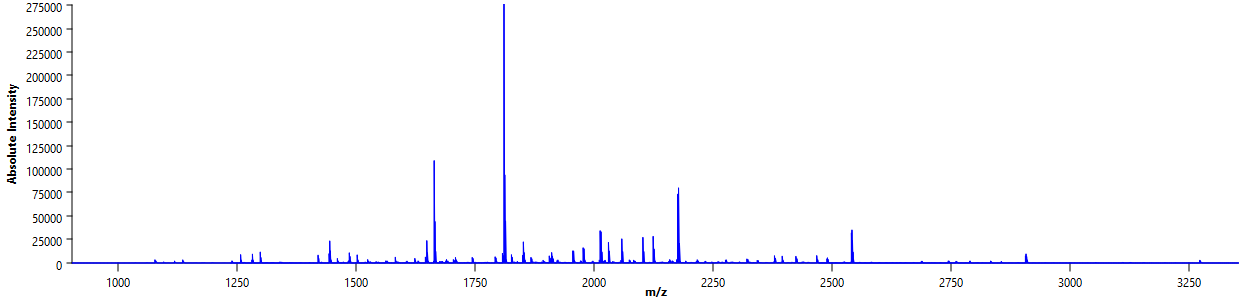
**Supplemental Figure 4. Spectra for MALDI-MSI of Glycans when MALDI-MSI is done in combination with MALDI-IHC.** MALDI-MSI was performed on both tissues on the same day using the same raster.

**MALDI-MSI First**

**MALDI-IHC First**

**Supplemental Figure 5. MALDI-MSI Spectra of collagen peptides when either MALDI-MSI or MALDI-IHC are performed first.** MALDI-MSI was performed on both tissues on the same day and analyzed with the same instrument method. Data are shown normalized to total ion current.

**MALDI-MSI First**


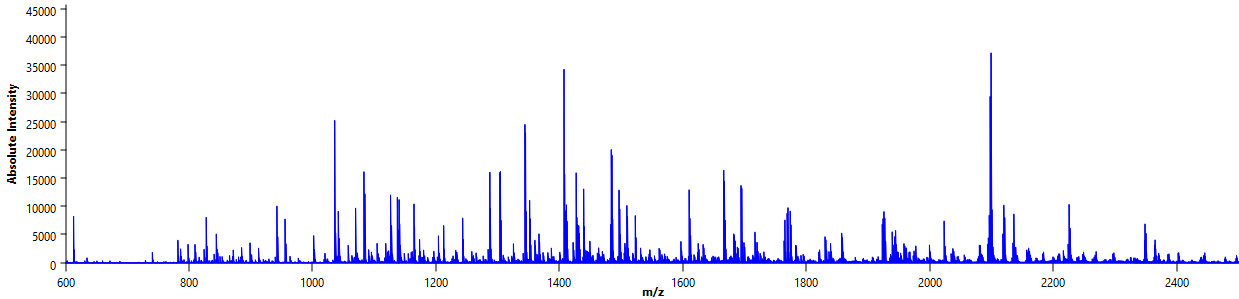

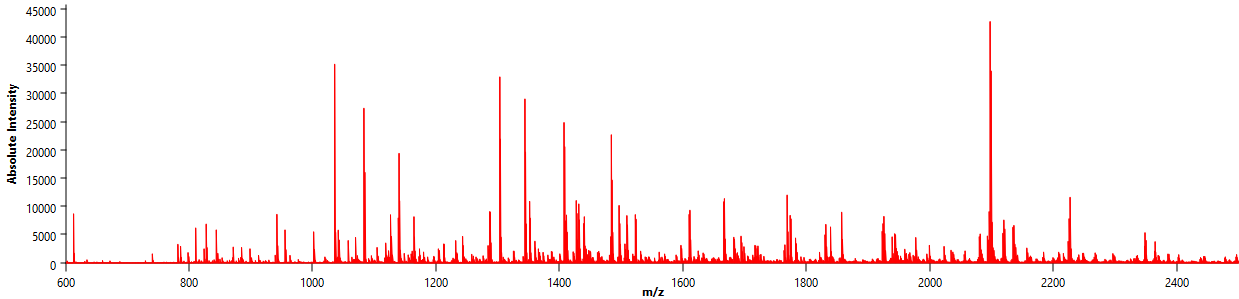


**MALDI-IHC First**

# GeoMx and MALDI-MSI

**Supplementary Figure 6.** **Spectra for Glycan Imaging when either MALDI-MSI or GeoMx is performed first.** Glycan imaging was performed on the same day using the same instrument methods. GeoMx first shows reduced signal intensity, which may be due to harsher heating conditions (70°C for 4 hours). Data are shown normalized to total ion current.

**MALDI-MSI First**

##
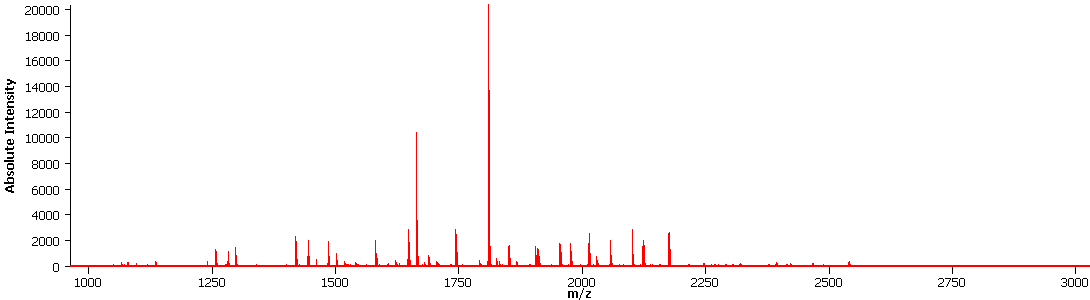


##
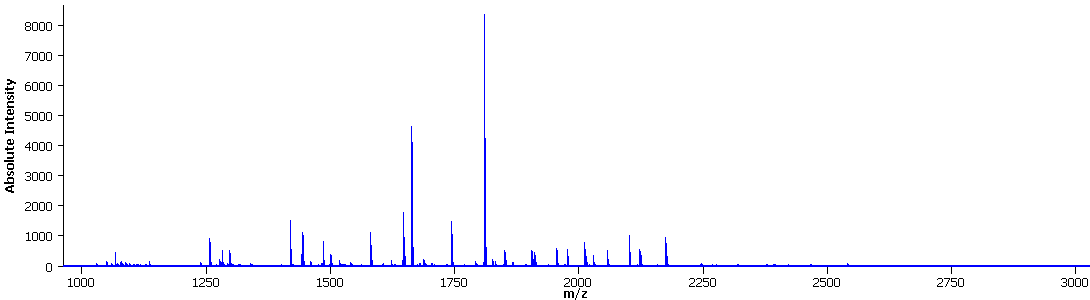


**GeoMx First**

**Supplementary Figure 7.** The decrease in N-glycan intensity seen in the breast TMA is reproduced in tonsil tissue. Spectra shown below demonstrate the differences seen in overall peak intensities. *Internal standard Glu-1-Fibrinopeptide B (GluFib) was added to the GeoMx first tissue, but not the MALDI-MSI first tissue.


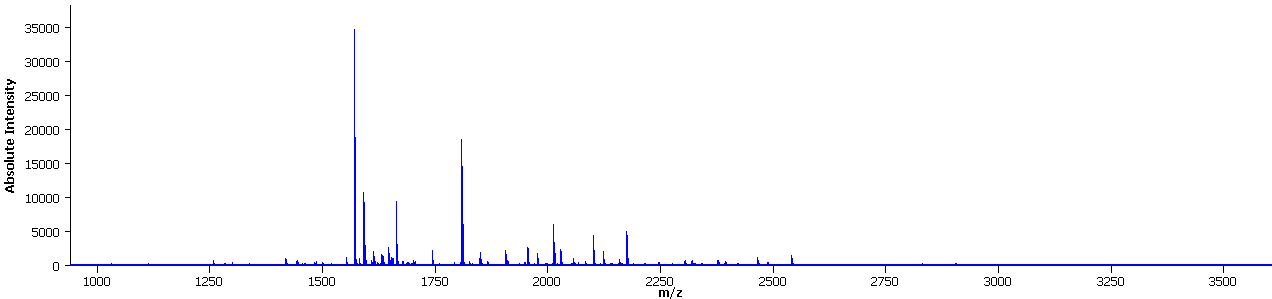

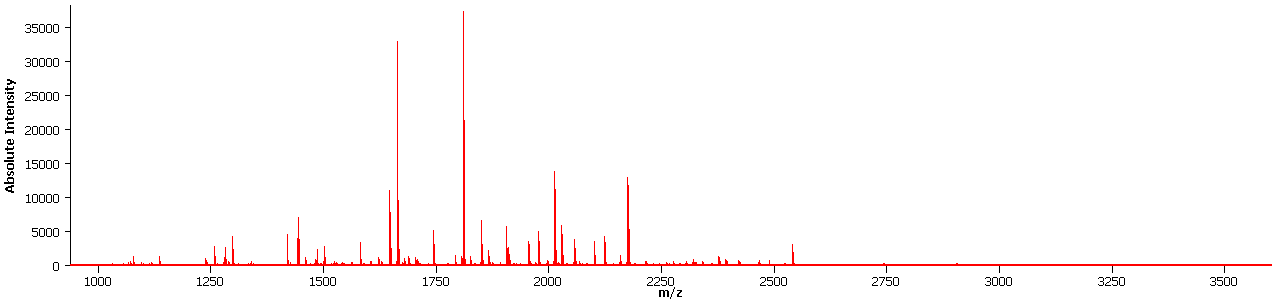

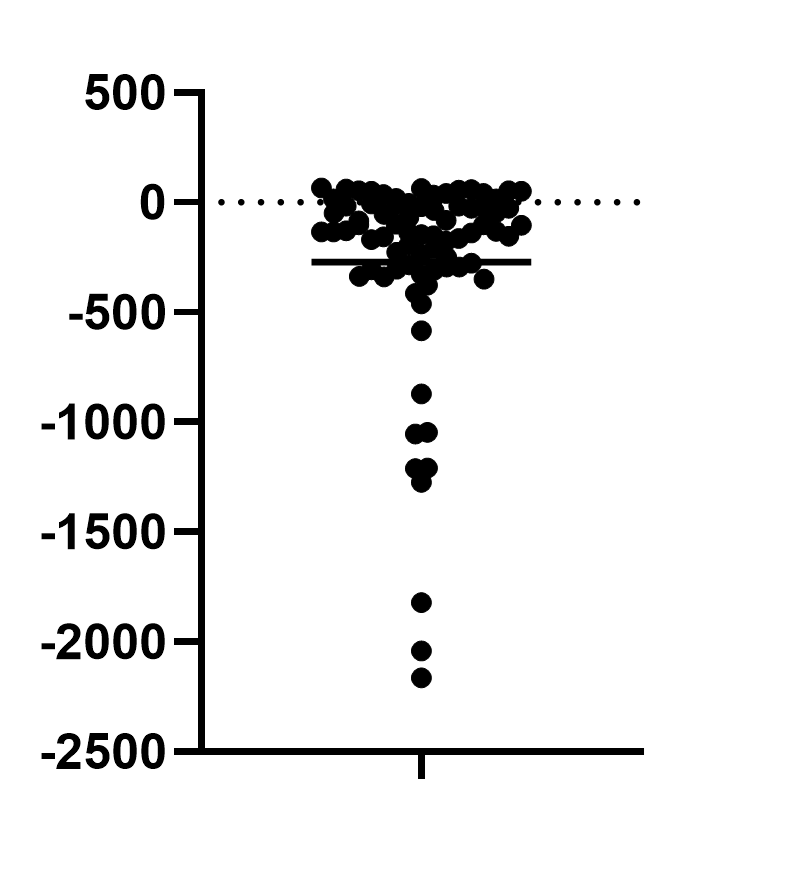

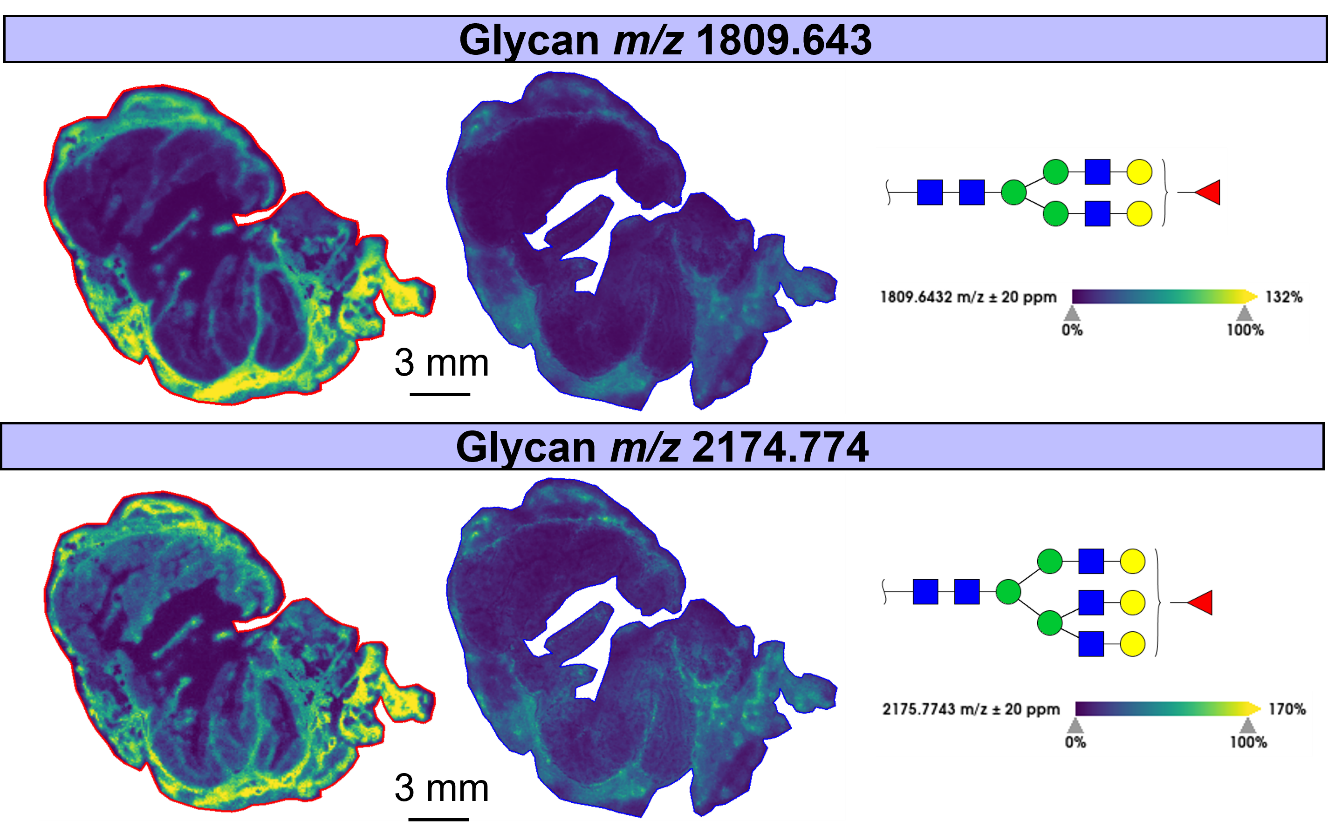


*****

**GeoMx First**

**MALDI-MSI First**

Difference in

peak intensity

Mean = -272.1

**Percent Change**

**Supplementary Figure 8.** **Spectra for ECM Imaging to compare spectral differences when either MALDI-MSI or GeoMx was performed first.** MALDI-MSI for both tissues was performed on the same day using the same instrument methods. Data are shown normalized to total ion current.

**
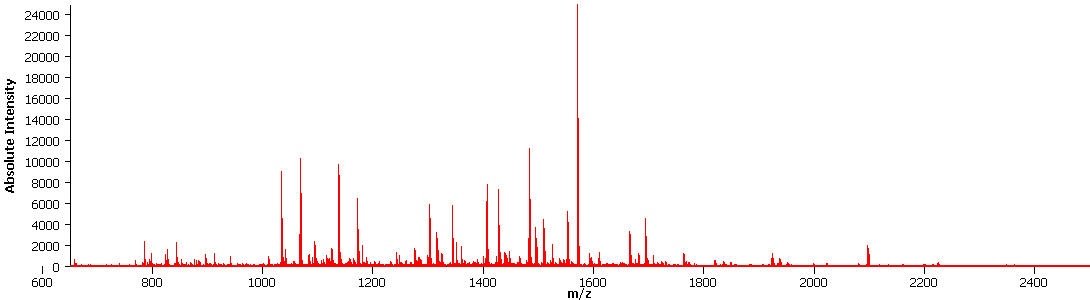
MALDI MSI First**

**GeoMx First**


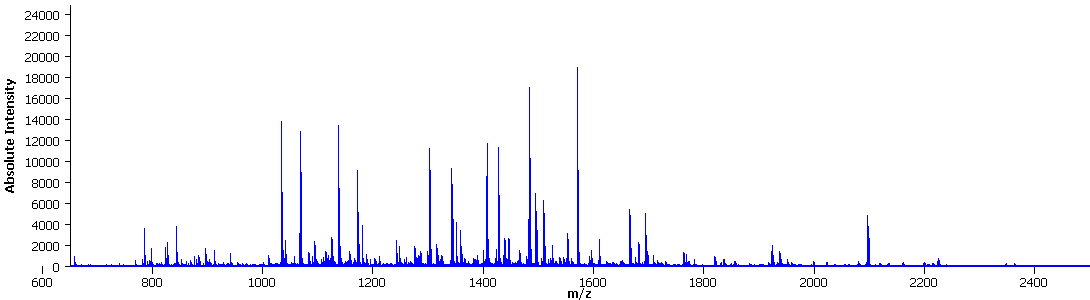


# Imaging Mass Cytometry

## Breast Cancer Imaging Mass Cytometry and MALDI-MSI


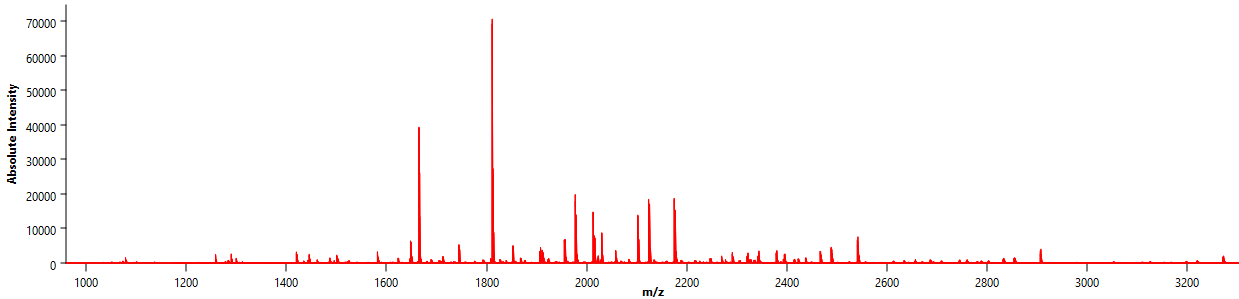
**Supplementary Figure 9.** **Spectra for Glycan Imaging on Breast Cancer tissues.** MALDI-MSI was performed on these tissues on the same day using the same instrument methods. Data are shown normalized to total ion current.

**MALDI-MSI First**


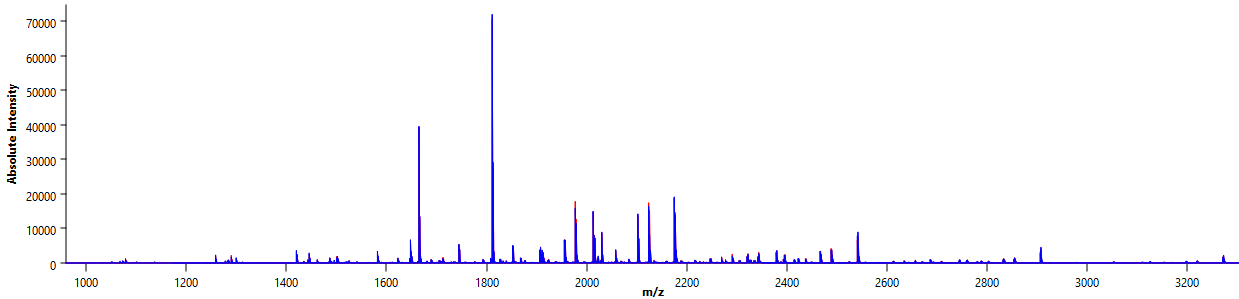


**IMC First**


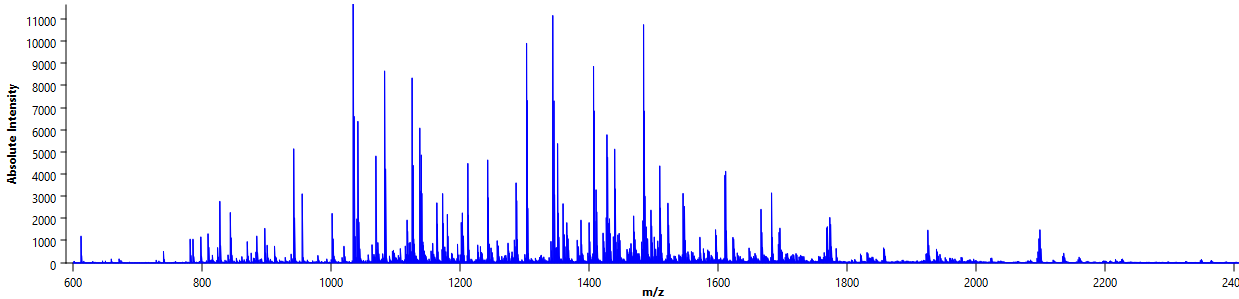

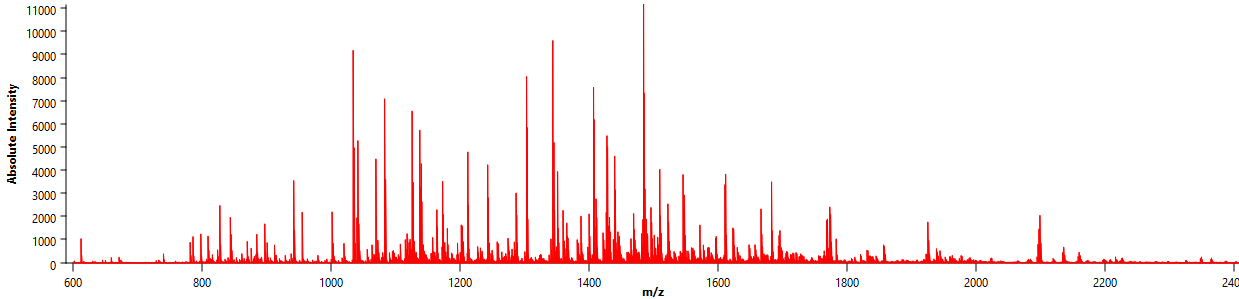
**Supplementary Figure 10.** **Spectra for Collagen Imaging on Breast cancer tissues.**  MALDI-MSI of collagen peptides was performed on the same day using the same instrument methods. Data are shown normalized to total ion current.

**MALDI-MSI First**

**IMC First**

## Liver Cancer Imaging Mass Cytometry and MALDI-MSI

**Supplementary Figure 11.** **Spectra for Glycan Imaging of Liver cancer tissues.** MALDI-MSI was performed on the same day using the same instrument methods for both tissues. Data are shown normalized to total ion current.

**MALDI-MSI First**


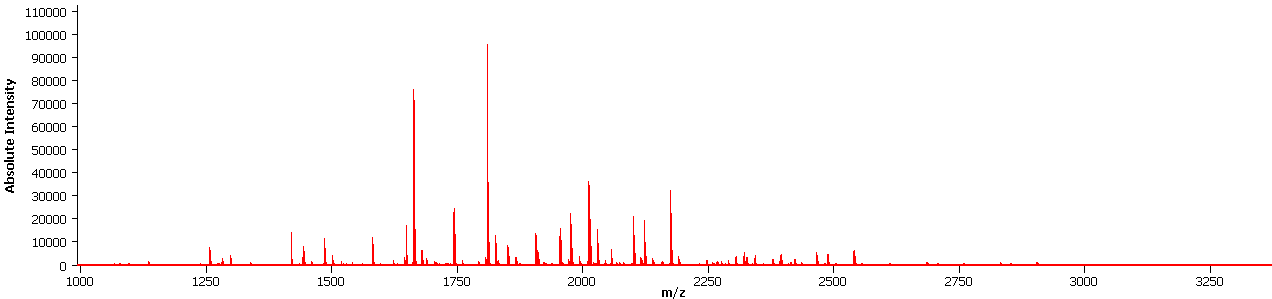


**IMC First**


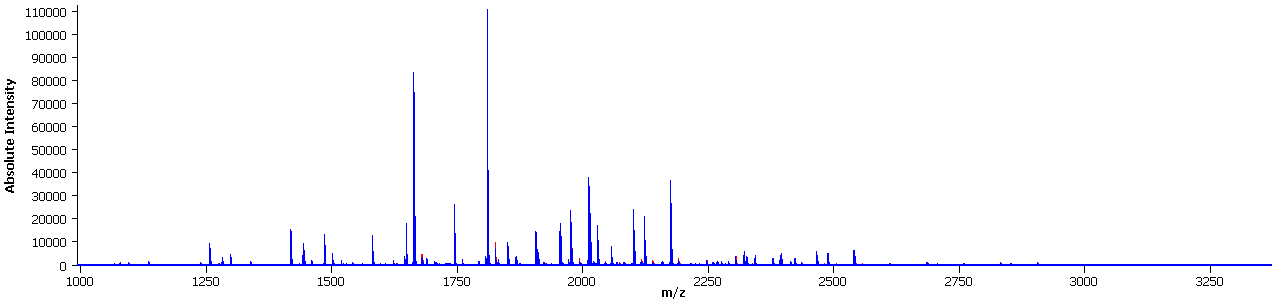


**Supplementary Figure 12.** **Spectra for Collagen Imaging of Liver cancer tissues.** MALDI-MSI for collagen peptides was performed on the same day using the same instrument methods. for each liver tissue section.

**
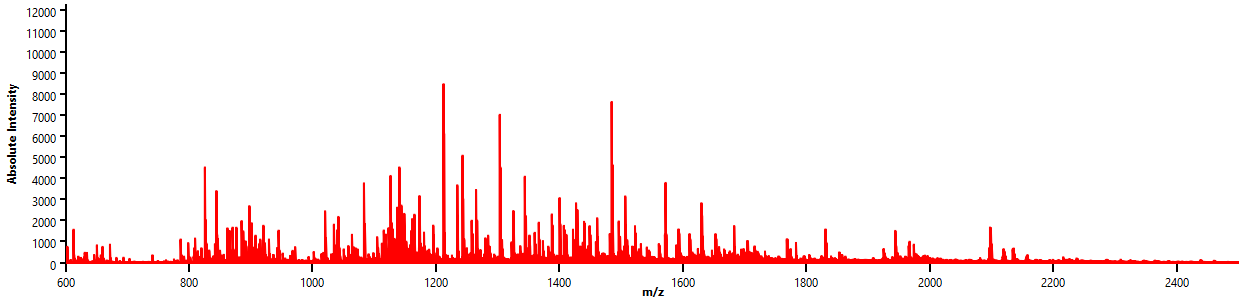
**

**IMC First**

**MALDI-MSI First**


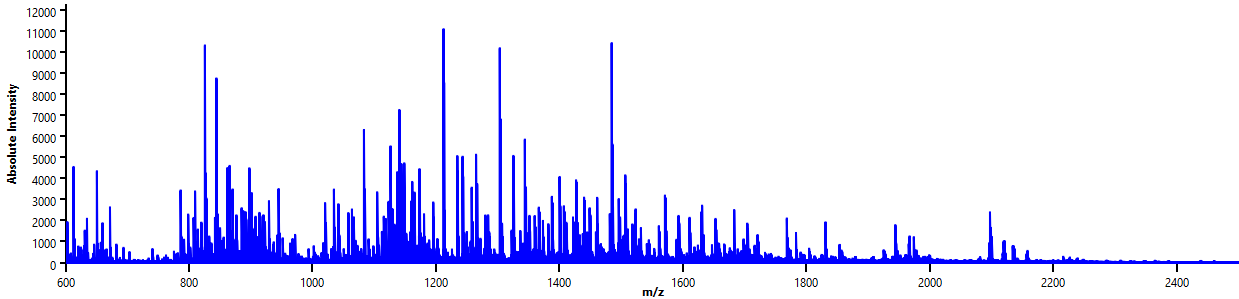


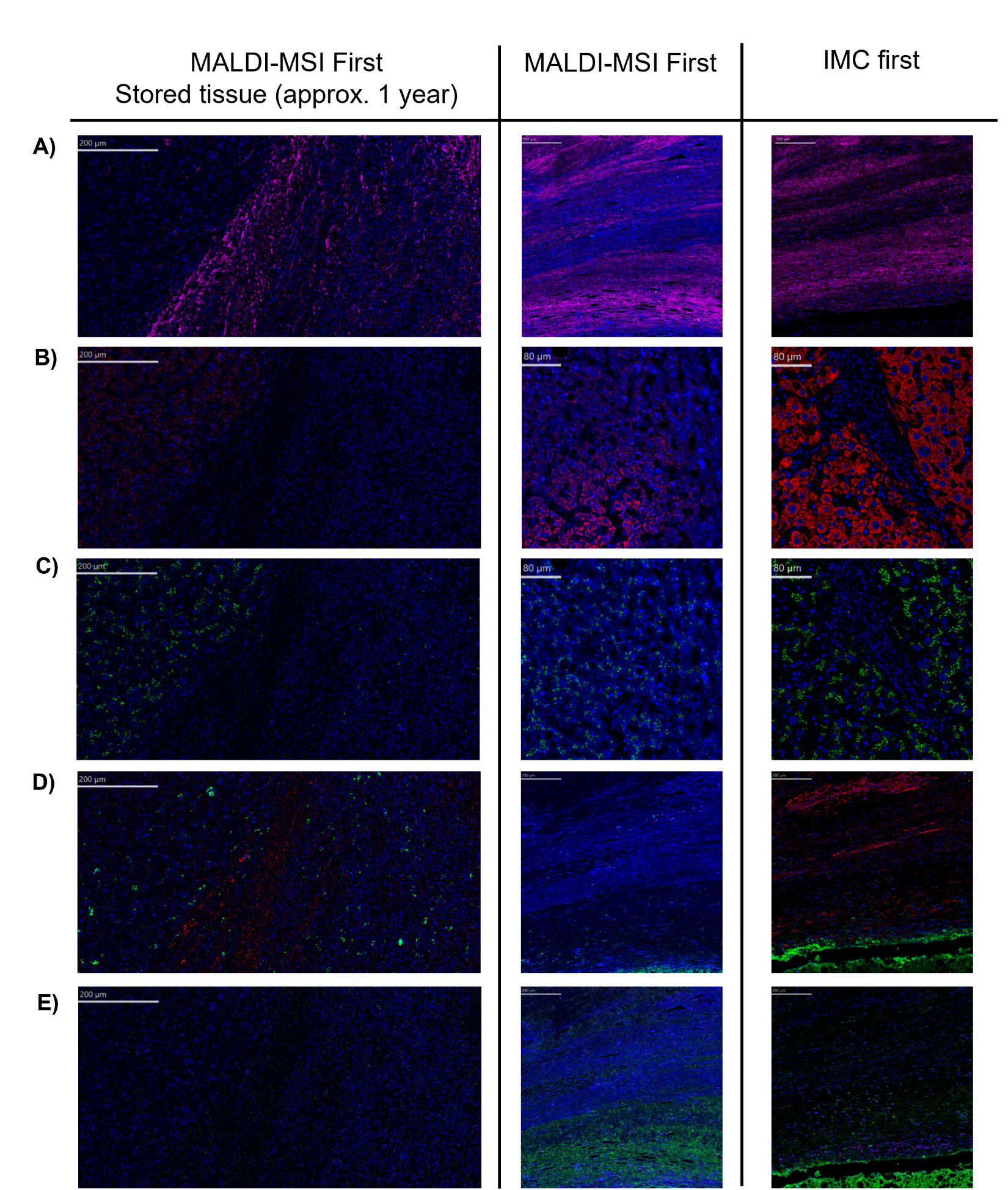


**Supplementary Figure 13. Imaging Mass Cytometry Images from 3 serial sections of liver tissue.** Tissues investigated include an older tissue processed by MALDI-MSI first (over 1 year old,left), a tissue processed by MALDI-MSI within days before CyTOF (middle), and a tissue processed by CyTOF only (right). In all images blue represents DNA and these colors represent the following markers in each row: A) magenta = collagen, B) red = HepPar1, C) green = STgal6, D) red = a SMA and green = CD68, E) magenta = CD45RO and green = OPN. While we do see some staining when MALDI-MSI is done first, the staining is the most robust when CyTOF is performed first.
